# Clinical Lectures on Diseases of the Lungs and Heart

**Published:** 1904-06

**Authors:** 


**Clinical Lectures on Diseases of the Lungs and Heart.** By James Alexander Lindsay, M.D. Pp. viii., 447. London: Baillière, Tindall & Cox. 1904.

The twenty-one chapters of this book are for the most part the substance of clinical lectures given by the author at the Royal Victoria Hospital, Belfast. They are intended to discuss the problems which confront the practitioner in the field of thoracic disease, more particularly those of diagnosis. Chapter v., on pleurisy, goes into the relation of pleurisy and tuberculosis, and the author gives a good practical summary, from which we learn "that an attack of acute pleurisy occurring in a previously healthy individual, and completely recovered from, is not a sufficient ground for apprehending the onset of pulmonary tuberculosis, provided the habits and mode of life are favourable to health; that such cases may be accepted for life policies at ordinary rates if two years have elapsed since the attack of pleurisy, and if the general health, pulmonary expansion, and weight are satisfactory." A very interesting chapter is No. VI., on the early diagnosis of pulmonary phthisis; it points out the folly of a dilatory policy by postponing the diagnosis until either bacilli are found in the sputum or definite physical signs detected in the lungs. The views of Dr. A. Béclère on the value of radioscopy in the diagnosis of incipient phthisis are given (p. 133), but the author himself awaits the verdict of a wider experience.

The increased hopefulness of prognosis in phthisis at the present day is the keynote of Lecture VIII., which gives an outline of the factors which influence it, and afterwards a series of typical cases in illustration.

In the lecture on treatment (No. IX.) the author states that his experience of tuberculin has been small but unfavourable: his extensive trial of antiseptic treatment has convinced him of its futility, and in recent years he has gradually discarded creasote and guaiacol, as he considers that the systematic use of remedies of very doubtful utility is to be deprecated. Accordingly he now rests content with hygienic and tonic measures without the aid of antiseptic drugs, and he considers that the hygienic treatment of phthisis rests upon the sound principle that, in the absence of a trustworthy specific for phthisis, the best means of combating the malady is to raise the general health to its highest power.

The last eight chapters have to deal with points of diagnosis and treatment of cardiac conditions, and in these the author shows himself to be an experienced and reliable guide.

